# Coping with drought: stress and adaptive responses in potato and perspectives for improvement

**DOI:** 10.3389/fpls.2015.00542

**Published:** 2015-07-22

**Authors:** Jude E. Obidiegwu, Glenn J. Bryan, Hamlyn G. Jones, Ankush Prashar

**Affiliations:** ^1^Cell and Molecular Sciences, The James Hutton InstituteDundee, UK; ^2^Plant Science Division, School of Life Sciences, University of DundeeDundee, UK; ^3^School of Plant Biology, University of Western AustraliaCrawley, WA, Australia

**Keywords:** potato, drought, yield, high throughput phenotyping, water use efficiency, breeding

## Abstract

Potato (*Solanum tuberosum* L.) is often considered as a drought sensitive crop and its sustainable production is threatened due to frequent drought episodes. There has been much research aiming to understand the physiological, biochemical, and genetic basis of drought tolerance in potato as a basis for improving production under drought conditions. The complex phenotypic response of potato plants to drought is conditioned by the interactive effects of the plant's genotypic potential, developmental stage, and environment. Effective crop improvement for drought tolerance will require the pyramiding of many disparate characters, with different combinations being appropriate for different growing environments. An understanding of the interaction between below ground water uptake by the roots and above ground water loss from the shoot system is essential. The development of high throughput precision phenotyping platforms is providing an exciting new tool for precision screening, which, with the incorporation of innovative screening strategies, can aid the selection and pyramiding of drought-related genes appropriate for specific environments. Outcomes from genomics, proteomics, metabolomics, and bioengineering advances will undoubtedly compliment conventional breeding strategies and presents an alternative route toward development of drought tolerant potatoes. This review presents an overview of past research activity, highlighting recent advances with examples from other crops and suggesting future research directions.

## Introduction

Potato (*Solanum tuberosum* L.) is of great economic value and ranks as the fourth most important food crop in the world. According to FAO (2011) global cultivation on 19.2 million hectares resulted in an estimated 374 million tons production. Potato production provides food, employment, and income as a cash crop (Scott et al., 2000) and helps in increasing food availability while contributing to a better land use ratio by raising the aggregate efficiency of agricultural production systems (Gastelo et al., 2014). Potatoes are grown in over 125 countries and more than a billion people worldwide consume them on a daily basis (Mullins et al., 2006). The poor and most undernourished farm households in many developing countries depend on potatoes as a primary or secondary source of food and nutrition (Lutaladio and Castaidi, 2009) as its short and flexible vegetative cycle makes it well suited for crop rotation with other major crops. Moreover, it can be cultivated in environmental conditions where other crops may fail (Food and Agriculture Organization of the United Nations, 2009). Global geographical production of potatoes shows that it is grown in all continents except Antarctica (Rowe and Powelson, 2002). Along with its flexibility for cultivation, potato also represents an excellent source of nutrients including carbohydrates, proteins, vitamin C, several forms of vitamin B, and minerals (Camire et al., 2009; White et al., 2009; Birch et al., 2012). As a major staple food, potato tubers are high in compounds including ascorbate, β-carotene, organic acids, and cysteine-rich polypeptides that promote mineral bioavailability. Furthermore, potato is low in anti-nutrients such as oxalates and phytates which can decrease mineral bioavailability (White et al., 2009).

Drought already poses one of the most important constraints to plant growth and terrestrial ecosystem productivity in many regions all over the world (Chaves et al., 2003) and water availability is becoming even scarcer for agricultural communities. The influencing factors include inadequate rainfall, excessive levels of salts in the soil solution or the increasing diversion of limited fresh-water resources to competing urban and industrial uses (Neumann, 2008). Climatic model predict that global warming will further escalate drought as a result of increasing evapotranspiration (Salinger et al., 2005; Cook et al., 2007) though there are likely to be large regional differences (Metz et al., 2007) with frequency and intensity increasing from 1 to 30% in extreme drought land area by 2100 (Fischlin et al., 2007). These negative consequences of climate change in agriculture will drastically affect poor and marginalized groups who depend on agriculture for their livelihoods coupled with limited potentials to cope. Impact of climate change on potato production in Ireland using simulation models (in the DSSAT package) showed an expected relatively uniform increase in temperature of about 1.6°C over the country by the 2075 climate period (Holden et al., 2003) and potato yield in 2055 and 2075 is expected to fall for non- irrigated tubers resulting in severe loss of yield over most of the country by 2055 (Holden et al., 2003). In northern temperate regions there will be more heterogeneity in weather events with northern Europe influencing more rain in winter and significantly less in summer (Haverkort and Verhagen, 2008) affecting both agriculture production and adaptation. In the Mediterranean and Sahel regions during the heat- free periods of the year potato yields will go down as the suitable periods become shorter. Higher evaporative demand will result to water being use less efficiently and only irrigation may hold the keys for yield increments in potato in the temperate (Haverkort and Verhagen, 2008). Globally, for potato, drought will decrease potential potato yield by 18–32% in the projected period of 2040–2069 (Hijmans, 2003). The indication for the effect of drought on potato production and cultivation can be seen from available FAO data and recent case studies. In 2011 and 2012 drought and heat waves in the USA have resulted in damage to summer crops (including potatoes) and livestock causing losses of $40–88 in billions of dollars (NOAA, 2011, 2012). In 2010, drought affected Russia and led to production losses of around 30% on industrial potato farms and households in the Central and the Volga Valley—a federal district that traditionally produces over 60% of the Russian potato crop (GAIN, 2010; Barriopedro et al., 2011). Crop models predict that potato yields may reduce by ~30% as a result of water deficit in Poland (http://www.climateadaptation.eu/poland/agriculture-and-horticulture/).

## Drought response

### Drought sensing and signaling

**Figure 1:**
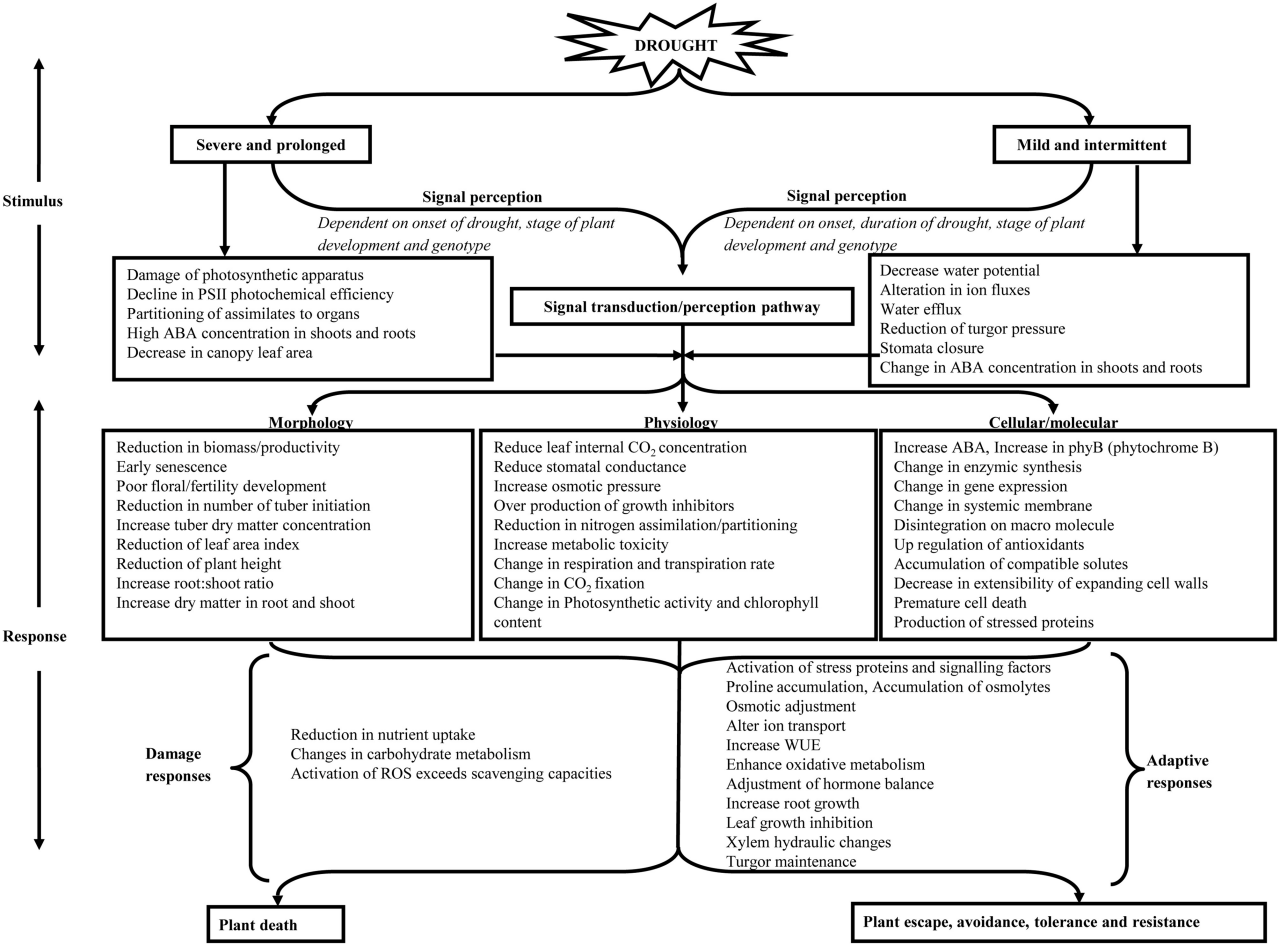
**Flow chart detailing the effect of different types of drought and how plants respond to the stimulus at molecular, physiological, and morphological levels**.

**Figure 2:**
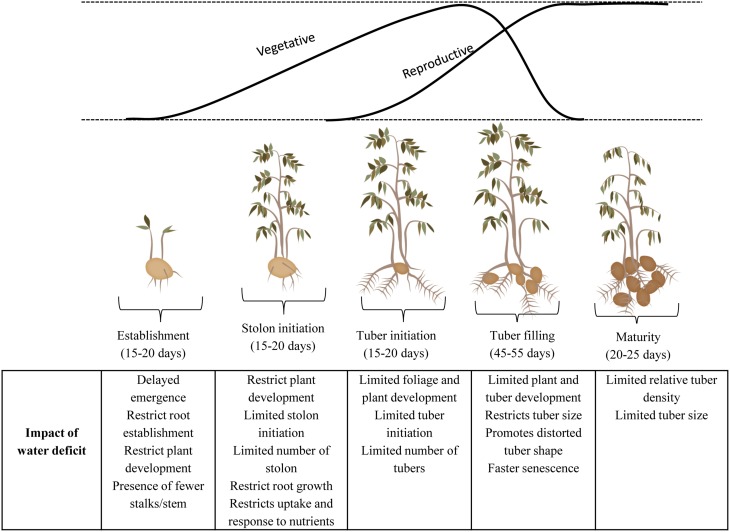
**Effect of water stress at different growth stages of potato**.

Drought limits productivity of crop plants by affecting photosynthetic processes at the canopy, leaf or chloroplast level, either directly, or by feedback inhibition if transport of photosynthate to sink organs is limited (Jones and Corlett, 1992). The response pattern of plants to drought is regulated by intensity, duration and rate of progression of imposed drought (Pinheiro and Chaves, 2011). Figure 1 provides an overview of the effect of different levels of drought and the response triggered under these levels. Under mild to moderate conditions, stomatal characteristics are affected which result in biomass loss while under severe conditions non-stomatal factors can become dominant (Liu et al., 2010) constraining photochemical efficiency and Rubisco activity and thus affecting the biochemical and physiological metabolisms (Xu et al., 2010). Light signal transduction pathway of guard cells remains the primary mechanism regulating stomatal opening (Lee, 2010), while mechanism of stomatal behavior is influenced by ABA in the root-to-shoot signaling under stress (Jia and Zhang, 2008). Plants grown under drought conditions tend to have lower stomatal conductance, thus helping to conserve water and maintain an adequate leaf water status but at the same time reducing leaf internal CO_2_ concentration and photosynthesis (Chaves et al., 2002). Nevertheless, the precise relationship is also dependent on other factors, for example genotype, influence of drought history, and environmental conditions (Schulze and Hall, 1982; Tardieu and Simonneau, 1998). It has been shown that long term exposure of leaf or canopy to low vapor pressure deficit generates a sort of “memory” in guard cells that results in loss of closing stimuli of stomatal responses (Nejad and Van Meeteren, 2007; Fanourakis et al., 2011). Thus, when studying drought response, differentiation is needed between terminal or intermittent drought and its interaction with environment. In the case of terminal drought the availability of soil water decreases progressively leading to premature plant death while intermittent drought comprises finite periods of inadequate water occurring at one or more intervals during the growing season (Neumann, 2008) (Figure 1). The timing of intermittent drought during growth may have much larger impact on biomass and yield than intensity of drought and may also depend on stress duration and phenological stage (Pinheiro and Chaves, 2011). Yield losses depend on plant growth phase affected by drought (vegetative and reproductive) (Serraj et al., 2004), which indirectly reduces the photosynthetic rate, CO_2_ fixation and finally resulting in less assimilate product (Mafakheri et al., 2010). Figure 2 details different growth stages in potato and how water limited environment can influence plant growth and development and eventually yield even if the lack of water is transient. In potato, tuber yield is correlated with both harvest index (HI) and dry matter and therefore for breeding, it is critical to understand the genotypic variation dependence on assimilate distribution and the dry matter production (Jefferies and Mackerron, 1987, 1993; Mackerron and Jefferies, 1988; Gregory and Simmonds, 1992; Jefferies, 1993a; Tourneux et al., 2003b). Little information is available on the effects of prolonged long term water shortage regimes but studies have widely examined short term lack of water consequences suggesting that soil moisture should not drop below 50% of crop available water in the soil for maximum yield (Mackerron and Jefferies, 1986). Drought in potato not only reduces yield, in that the crop may extract less of the available water from the soil in comparison with other crops (Weisz et al., 1994), but it can also harm product quality, for example by increasing common scab incidence (Mane et al., 2008).

At the molecular and biochemical level, plants perceive and respond to drought stress by dynamically shifting regulatory responses during transcription and protein expression, thus affecting many biochemical pathways and consequently physiological and developmental processes (Mane et al., 2008; Vasquez-Robinet et al., 2008). The reduction in photosynthesis as a result of drought is mediated through the stomatal and non-stomatal effects, where later involves decreased electron flow as a result of both photo-inhibitory damage and regulatory control of energy dissipation in the chloroplasts (Angelopoulos et al., 1996; Baker, 2008). Stomatal closure decreases CO_2_ availability in the mesophyll, and changes in the electron transport and biochemical pathways (Boyer, 1976) all contributing to reduced photosynthesis (Cornic et al., 1983; Genty et al., 1987) under water deficit stress. Biochemical changes include decreased ribulose bisphosphate (RuBP) synthesis (Gimenez et al., 1992), decreased Rubisco activity and decreased carboxylation efficiency (Martin and Ruiztorres, 1992) or both (Faver et al., 1996). The progressive decrease in maximum metabolic capacity under stress saturates photosynthetic rate and thus suggests, implicates photosynthetic carbon reduction cycle enzymes, including RuBP carboxylase/oxygenase (Rubisco). Limitation of RuBP synthesis is probably caused by inhibition of ATP synthesis, due to progressive inactivation or loss of coupling factor resulting from increasing ionic (Mg^2+^) (Lawlor, 2002). Selective enhancement of Rubisco activase capacity could partly enhance photosynthesis under stress, but this enhancement would generally be small (Parry et al., 2013). At the electron transport level, decreased CO_2_ assimilation leads to reduced regeneration of NADP+ in the Calvin cycle (Schapendonk et al., 1989; Cruz de Carvalho, 2008) resulting in concomitant generation of reactive oxygen species (ROS) and potential photo-inhibitory damage. Moreover, decreased intercellular CO_2_ concentration (as stomata close) favors oxygenation of RuBisCO which can also contribute to ROS production (Cruz de Carvalho, 2008). Figure 1 provides a detailed description of plant response to drought, stimulus to different types of drought, their effect on signaling pathways and plant adaptive responses. Different signaling pathways initiate phosphorylation cascades and activate appropriate transcription factors which trigger cellular responses under drought. These transcription factors regulate gene expression as a primary response of the plant resulting in plant stress adaptation (Rensink et al., 2005). Cytosolic Ca^2+^ is implicated as a cellular sensor second messenger in signal transduction, intracellular communication, and coordination of parts of recipient cells toward a behavioral objective (Sanders et al., 2002; Trewavas, 2002). In addition to localized responses there is compelling evidence for an important role for long-distance signaling, for example, abscisic acid (ABA) as a major chemical root to shoot stress signal in plants grown under dry conditions (Davies and Zhang, 1991; Davies et al., 2002; Dodd, 2005; Hey et al., 2010). As a response to drought signal, the dosage of plant hormone ABA rises relative to stress severity and it represents a key signal in cellular response by activating the expression of different drought responsive genes (Chaves et al., 2003). Other hormonal signals sent to the shoot through transpiration stream along with ABA upon drought perception include ethylene and Gibberellic acid (GA). Studies have revealed that these hormones invoke responses in both mature and young leaves, with ABA response in mature leaf cells and ethylene and GA response in dividing and expanding leaf cells (Skirycz et al., 2010; Verelst et al., 2010). Similarly, Jasmonic acid (JA) or Jasmonates are shown to be important cellular regulators activating signal transduction pathway between stress perception and response under drought conditions (Wasternack and Parthier, 1997; Du et al., 2013; Lee et al., 2013). Some protein families serve as an interface of ABA, JA, and (GA) signaling, suggesting a pivotal functional involvement of lipid-derived signaling in abiotic stress responses (Golldack et al., 2014). Compatible solutes including amino acids, ammonium compounds, sulfonium compounds, sugars, and polyols protect cellular structures (Chen et al., 2007). To protect plant enzymes and proteins from denaturizing upon drought stress, compatible solutes help to stabilize enzymes (Büssis and Heineke, 1998). The differential distribution and accumulation response of plants to this complex interaction defines their status toward survival or death. Plant cell death under severe drought stress occurs when concentration in ROS exceeds the scavenger potential with a consequence of irreparable damage to different plant cells including lipids, proteins, and deoxyribonucleic acid (DNA).

### Adaptive response

Plants respond to limiting water availability through a complex series of adaptive changes often accompanied by deleterious pleiotropic effects (Chaves et al., 2002; Jones, 2014). In an agricultural context farmers and breeders tend to define drought tolerant cultivars as those that maintain yield under drought conditions. Water deficit elicits responses that are expressed on growth, reproduction, partitioning of assimilates, survival, and death. These adaptive responses can be short term physiological regulation, cellular reversible adjustments, structural irreversible adjustments, and genotypic adjustments. The evolutionary responses to water stress are seen in light of genetic adaptation which produces specialized set of traits that allows them to prevail (Körner, 2003). Adaptation of plants to drought can involve avoidance or tolerance and thus stress adjustment scenarios that can be partitioned into (i) avoidance of tissue water deficits/dehydration, (ii) tolerance of tissue water deficits, and (iii) efficiency mechanisms (Turner, 1986; Jones, 2014). Avoidance of tissue water deficits (drought resistance according to Levitt, 1980) can be achieved by means of “drought escape,” where a plant grows only during periods of ample water availability and often involves rapid phenological development. The drought escape process is significant in arid regions where adapted annuals might combine short life spans with high rates of growth and gas exchange while utilizing the maximum moisture content in soil (Maroco et al., 2000). Avoiding tissue dehydration can also be achieved by enhanced water uptake associated with adaptive aspects such as increased root depth, altered rooting patterns (Jackson et al., 2000) or by reduced water loss as a result of mechanisms involving stomatal closure or adjustments to the leaf energy balance through reductions in light absorption or modifications to heat and mass transfer in the leaf boundary layer (Larcher, 2000; Mitra, 2001; Jones, 2014). High stomatal resistance can be observed when potato leaf temperature is above 25°C while vapor pressure gradient is relatively constant (Ku et al., 1977) resulting to significant reduction in transpiration. The closure of potato stomata under high leaf temperature is a reflection of adaptation to a cooler environment (Ku et al., 1977). Notably, the increased stomatal resistance may not result to proportional decrease in transpiration rate because for a given reduction in transpiration due to stomatal closure, the increase in leaf temperature would depend strongly on environmental factors, particularly the radiation load on the leaf, and the heat transfer coefficient of the air (Hsiao, 1973). Non-stomatal factors in the leaf, often referred to as “mesophyll,” cause significant reductions in transpiration as water stress develops. In addition, water deficit affects stomata via its effect on ABA levels or on plant hormonal balance (Chaves, 1991).

Although stomatal density and stomatal size play an important role in determining the transpiration rate and leaf conductance and especially providing a rapid response to water deficits, much of the most important control of water loss over the life cycle of a crop is achieved by adjustments to leaf area (Wolfe et al., 1983; Jones, 2014). Tolerance of tissue water deficits most commonly involves maintenance of turgor, even when the tissue water potential declines, either through osmotic adjustment (OA) (Morgan, 1984) or as a result of the presence of rigid cell walls or decreased cell size (Wilson et al., 1980). The substantial differences between species in their ability to tolerate tissue water deficits and thus continue metabolism at low water potentials is often associated with the build-up of so-called “compatible” solutes. Our functional definition of drought tolerance is based on combination yield stability and high relative yield under water deficits. This has been proposed as useful selection criterion for adaptation under varying degree of water deficit (Pinter et al., 1990). Potential efficiency mechanisms for improvement of crop drought tolerance include improvements in the water use efficiency (WUE) and improvements in the efficiency with which assimilate is converted to harvestable yield (HI). In practice a plant may combine a range of drought tolerance mechanisms (Ludlow, 1989), but it is important to note that there is an important trade-off involved with many “drought tolerance” mechanisms as they may reduce potential yield: for example stomatal closure conserves water but also reduces photosynthetic assimilation.

Understanding the interactions between the various biochemical, molecular and physiological mechanisms for stress adaptation is vital for identifying traits that might improve stress tolerance in crops through both conventional breeding and transgenic strategies (Evers et al., 2010). In the context of climate change, there is also an urgent need to understand and develop potato management practices that will help adaptation to water deficit and their interaction with traits that confer tolerance or resistance to stress. This review discusses the current state of knowledge in trait identification with evidence of our understanding of drought tolerance in potato and further highlights the strategies and mechanisms being used to address adaptation. The review also discusses the use of new “pheno-technological” platforms and how they can be used to narrow the gap between genotyping and phenotyping for drought stress and to enhance breeding practices.

## Drought effects on potato

### Physiological and morphological responses

Physiological responses are variety dependant and vary with source of the seed or physiological age of the tubers (Steckel and Gray, 1979). The differences in source may influence patterns of foliage growth and yield as well as the number of root primordia and the eventual size of root system (Wurr, 1978). As might be expected, physiological responses to drought are often more closely associated with “predawn” water potential than with daytime water potentials (Jones, 2007). Physiological response strategies include drought sensitive stomata which result in low transpiration rates and relatively increased intrinsic WUE regardless of radiation level and thus promote growth in areas with limited water availability (Coleman, 2008). Transpiration is affected by leaf area, root to leaf ratio, leaf orientation, leaf shape, leaf surface characteristics (pubescence), leaf thickness, and distribution of stomata. An overview of the studies highlighting the impact of drought on morphological/physiological traits in potato is shown in Table 1 (with guidance from Figure 1) and the crop stages at which these characters are influenced are highlighted in Figure 2.

**Table 1:** **Summary of drought impact on different morphological and physiological traits and summarized literature search highlighting these effects in potato**.

**Morphological/physiological traits**	**Decreasing references**	**Improving references**
Productive foliage (stem and leaf)	Jefferies and Mackerron, 1987, 1993; Fasan and Haverkort, 1991; Shock et al., 1992; Dallacosta et al., 1997; Deblonde et al., 1999; Albiski et al., 2012	−
Stem thickness	Albiski et al., 2012	
Plant dry matter	Jefferies and Mackerron, 1987; Jefferies, 1992; Albiski et al., 2012; Fleisher et al., 2013	Jefferies, 1993a; Jefferies and Mackerron, 1993
Canopy coverage	Jefferies and Mackerron, 1993	−
Leaf dry mass, leaf area index (LAI), leaf area duration (LAD	Fasan and Haverkort, 1991; Lahlou et al., 2003; Albiski et al., 2012	−
Leaf growth	Jefferies and Mackerron, 1993; Weisz et al., 1994	−
Leaf water potential	Moorby et al., 1975; Haverkort et al., 1991; Heuer and Nadler, 1998	−
Diffusive leaf resistance	−	Ierna and Mauromicale, 2006
Leaf osmotic potential	Heuer and Nadler, 1998	−
Leaf sugar concentration	−	Moorby et al., 1975
Number of green leaves	Fasan and Haverkort, 1991; Deblonde and Ledent, 2001	−
Plant water content	Albiski et al., 2012	−
Plant Height	Fasan and Haverkort, 1991; Shock et al., 1992; Deblonde and Ledent, 2001; Albiski et al., 2012	−
Tuber yield	Jefferies and Mackerron, 1987, 1989; Miller and Martin, 1987; Lynch and Tai, 1989; Spitters and Schapendonk, 1990; Fasan and Haverkort, 1991; Jefferies, 1992; Martin et al., 1992; Shock et al., 1992, 1998; Karafyllidis et al., 1996; Dallacosta et al., 1997; Steyn et al., 1998; Deblonde and Ledent, 2001; Lahlou et al., 2003; Tourneux et al., 2003b; Proietti et al., 2005; Ierna and Mauromicale, 2006; Shiri-E-Janagard et al., 2009; Evers et al., 2010; Ierna et al., 2011; Alva et al., 2012	−
Tuber dry matter	Levy, 1983; Jefferies and Mackerron, 1989, 1993; Deblonde et al., 1999	Steckel and Gray, 1979; Jefferies and Mackerron, 1987, 1993; Jefferies, 1992; Nadler and Heuer, 1995
Number of tuber	Mackerron and Jefferies, 1986; Lynch and Tai, 1989; Martin et al., 1992; Shock et al., 1992; Deblonde and Ledent, 2001; Lahlou et al., 2003; Onder et al., 2005	−
Tuber specific gravity	Shock et al., 1993, 2003; Eldredge et al., 1996	
Stem-end reducing sugar	−	Levy, 1983; Shock et al., 1993; Nadler and Heuer, 1995; Eldredge et al., 1996
Harvest index	Fasan and Haverkort, 1991	Fleisher et al., 2013
Stolon number	Haverkort et al., 1990	Lahlou and Ledent, 2005
Tuber quality at storage	−	Shock et al., 1992
Total soluble solids	Levy, 1985	Shock et al., 1993
Tuber osmotic potential	Levy, 1985	−
Partitioning of assimilate into tubers	−	Jefferies and Mackerron, 1989
Root length	Albiski et al., 2012; Auber et al., 2013	Jefferies, 1993a
Root number and thickness	Albiski et al., 2012	−
Root biomass	Mane et al., 2008	−
Root water potential	Liu et al., 2005	−
Root dry matter	−	Jefferies, 1993a; Lahlou and Ledent, 2005
Root: shoot ratio	−	Jefferies, 1993a

Plants under water stress show a decrease in leaf conductance (g_1_) which largely declines in parallel with leaf water potential (Ψ_1_) with efficient water conservative strategy dependant on the characteristics of canopy architecture such as canopy area, open/close canopy, leaf orientation, and cuticular transpiration rates. Potatoes exhibit isohydric characteristic with soil water potential (Ψ_soil_) and stomatal conductance (g_s_) decreasing under water stress while maintaining Ψ_1_ similar to values obtained from non stressed conditions (Liu et al., 2005) suggesting delay in onset of stress. Predawn Ψ_1_ and g_s_ can therefore be used as parameters for water stress as they exhibit coherent relationship with growth and yield In potato, water stress studies have shown reduction in expansion of stems and leaves, leading to reduced foliage, reduced canopy, reduced leaf area index, decreased shoot biomass, and finally reduction in dry matter content (Jefferies, 1992, 1993b; Jefferies and Mackerron, 1993; Dallacosta et al., 1997; Deblonde et al., 1999; Deblonde and Ledent, 2001; Mane et al., 2008; Albiski et al., 2012; Anithakumari et al., 2012). These morphological responses depend on the time of stress (development stage) and also whether the stress is short or long term. Stress at stolon initiation and tuber initiation stages not only restrains foliage and plant development but also limits the number of stolons leading to reduced tuber numbers and therefore, reduced HI and dry matter with effects on final yield (Fasan and Haverkort, 1991; Deblonde and Ledent, 2001; Lahlou et al., 2003; Tourneux et al., 2003b; Evers et al., 2010). In some cases, studies have shown some contrasting effects on the morphological and physiological traits, which can be attributed to genotype x environment interaction. Contrasting reports of increased HI (Fleisher et al., 2013) and reduced HI (Fasan and Haverkort, 1991) can be credited to varying enviromental conditions used for the studies. A controlled sunlit soil–plant-atmosphere research (SPAR) chamber with short term drought cycles was used in the former while the latter were grown under a permanent rain shelter in mobile containers. Root length has been reported to decrease under water stress using both *in vitro* screening (Albiski et al., 2012) and terminal drought evaluation (Auber et al., 2013). This contrasts with an earlier report (Jefferies, 1993a) that showed a constant root length while using self- and reciprocal grafts as planting materials with scions having a dominant effect in determining the partitioning of dry matter between shoot, root and tubers. Studies by Katerji and colleagues reported significant decrease in WUE when crops are grown in clay soil as during the active growing phase, a reduced water uptake occurs in the plants growing in the clay soil (Katerji and Mastrorilli, 2009) contradicting the results showing WUE not affected by soil type and salinity (Karam et al., 1996). Similar contradictory results have been reported for stolon numbers, with drought either reducing the stolon number (Haverkort et al., 1990) for a series of experiments in plots, in pots or in containers in a glasshouse or under a rain shelter environment or increasing the number (Lahlou and Ledent, 2005) for other experiments under field and green house conditions. In all these contrasting studies, the environmental conditions, soil type, stress severity and phenological stage of stress induction differed and may have led to the variability in results.

Plant size, reduced leaf area, early maturity, and prolonged stomata closure are key traits in response to mitigating the effect of drought on plants (Weisz et al., 1994; Mahan et al., 1995; Karamanos and Papatheohari, 1999; Farooq et al., 2009; Xu et al., 2009). Similarly, the ability of potato to form a large “above ground” biomass has been shown to be an effective insurance against soil water deficit (Schittenhelm et al., 2006). As discussed above, plant response to drought may involve avoidance or tolerance. While avoidance and resistance responses involves morphological restraints, change in canopy size, area and anatomy and increase stomatal and cuticular resistance, tolerance is primarily attributed to maintenance cell turgor that includes OA and cellular or tissue elasticity. The limitation in measuring large number of genotypes for traits linked to tolerance without high throughput approaches has led to the use of transgenic approaches to bioengineer crop plants for tolerance (discussed in next Section).

Crop growth models allow us to evaluate the complexity of drought tolerance mechanisms and should help to provide a basis for identification of optimal strategies for any specific environmental combination. For example, the closing of stomata early in response to drought can save water for future growth at the expense of current growth but without affecting final growth and assimilate product (Spitters and Schapendonk, 1990). Although work is still being carried out to understand the physiological basis for this association, carbon isotope discrimination (δ^13^C) shows a significant positive correlation with plant height and the dry biomass of the plant foliage (Legay et al., 2011; Anithakumari et al., 2012). δ^13^C measures the ratio of stable carbon isotopes (^13^C/^12^C) in the plant dry matter compared to the ratio in the atmosphere (Condon et al., 1990). In a water deficit condition, δ^13^C represents a reliable estimator of stomatal conductance (Condon et al., 2002) and WUE in crops (Turner, 1997; Tambussi et al., 2007). Studies show that drought tolerant genotypes exhibited high WUE, stomatal control, and root elongation by maintaining photosynthesis and putative sucrose export to tubers under drought exposure. Studies show that high CO_2_ assimilation rate favors high N assimilation which in turn up-regulates nitrate reductase activity (NR), with lower decrease in NR associated with drought tolerance (Balasimha and Virk, 1978; Das et al., 2005).

### Drought responsive genes

Stress responsive expression is thought to be an important mechanism of adaptation as it plays an important role in tolerance. Several studies have cataloged a large number of genes showing differential expression during stress treatment both in cereals and model crops. Although this list can be differentiated into WUE, avoidance and escape and finally tolerance based on traits associated, this paragraph compiles them under one heading as these mechanisms are interactive and response of one pathway affects the other. Studies have shown that glycolysis gene transcription and amino-acid degradation are more strongly repressed in drought tolerant cultivars when compared to drought susceptible ones (Evers et al., 2010). Drought stress led to strong repression of photosynthesis-related genes in drought tolerant cultivar (Reiter and Vanzin, 2001; Evers et al., 2010). Under stress recovery, genes associated with PSII showed that genotype specific activation responses increased sucrose accumulation and cell wall biosynthesis (Mane et al., 2008). There is evidence of high mitochondrial metabolic activity in a drought resistant cultivar (Vasquez-Robinet et al., 2008) while a susceptible cultivar was characterized with higher levels of proline, trehalose, and GABA (γ-aminobutyric acid) (Vasquez-Robinet et al., 2008). Several genes associated with mitochondrial function were more negatively affected in drought susceptible genotypes. Dihydrolipoamide dehydrogenase and ADP-sugar biphosphatase were activated in drought resistant clones but repressed in drought susceptible clones. Chloroplast-localized antioxidant and chaperone genes were more highly expressed in drought resistant cultivars whereas ABA-responsive transcriptional factors (TFs) were more highly expressed in drought susceptible ones (Vasquez-Robinet et al., 2008). Evaluation under *in vitro* control and water-stressed conditions identified putative candidate genes for drought tolerance as transcription factors and signaling molecules, such as protein kinases and ERF1 (Ethylene Response factor 1) (Anithakumari et al., 2011, 2012). Silencing of a CBP80 (nuclear cap-binding protein) gene in the cultivar *Desiree* increases water deficit tolerance through initiating Cap-Binding Complex (CBC) that recognizes and binds to the cap structure of RNA polymerase II transcripts in the nucleus (Papp et al., 2004; Kierzkowski et al., 2009; Pieczynski et al., 2013). The drought tolerant plant silenced for the *CBP80* gene exhibited an ABA-hypersensitive stomatal closing response (Pieczynski et al., 2013). Products of *miR*159, *MYB33*, and/or *MYB101* genes that act downstream of *CBP80* have been shown to be involved in the ABA- mediated regulation of potato responses to drought and similar studies have also identified and characterized microRNA families for drought stress response and their putative target genes including miR171 (stu-miRNA171a, b, and c), miR159, miR164, miR166, miR390, miR395, miR397, miR398, miR408, and miR482 (Hwang et al., 2011a,b; Pieczynski et al., 2013; Zhang et al., 2013). Most of the genes identified in the above studies showing differential down regulation were involved in photosynthesis and carbohydrate metabolism, including chlorophyll a-b binding proteins, fructose-1, 6-bisphosphatase trehalose-6-phosphate synthase while sucrose synthase was up-regulated (Hwang et al., 2011a,b; Kondrak et al., 2012).

Changes in transcript profiling upon imposition of polyethylene glycol (PEG) induced water stress show that up-regulated genes were prevalently involved in carbohydrate metabolism, cellular communication, and signal transduction whereas down regulated genes mostly include ATP-dependent RNA helicase and cytochrome P450 followed by vacuolar ATP synthase and genes involved in protein synthesis (Leone et al., 1994; Ambrosone et al., 2011; Zhang et al., 2014). Genes involved in different pathways of protein metabolism participate in the early response, controlling protein folding, and protein turn-over/degradation thus contributing to regulation of protein synthesis while preventing major cellular damage under water deficit (Ambrosone et al., 2013). Table 2 shows the list of differential gene expression using validated RT-PCR and microarray using leaf material under water and PEG induced stress in potato. Dehydrins (group 2 members of late embryogenesis abundant protein family) are shown to be associated with crucial protective functions through membrane stabilization, resistance to osmotic pressure and protection of proteins—the so-called chaperone function (Allagulova et al., 2003; Lopez et al., 2003; Agoston et al., 2011). Studies show that the dehydrins interact with membranes in the interior of cells and reduce dehydration induced damage through their ability to replace water and, through their hydroxyl groups (Baker et al., 1995) and dehydrins are shown to play a fundamental role in plant response and adaptation to abiotic stress (Hanin et al., 2011). Comparing expression analysis in non-transgenic potato and AtDREB1A (Dehydrin responsive element binding protein) transgenic potato suggest that potato may have mechanisms in abiotic stress tolerance controlled by native TFs similar to AtDREB1A (Watanabe et al., 2011). The over expression of *StMYB1R-1* (MYB-like transcription factor) improved plant tolerance to stress and it is thought that the induced upregulated expression of AtHB-7 (Arabidopsis homeobox gene), RD28 (Responsive to desiccation), ALDH (Aldehyde dehydrogenase), and ERD15 (Early response to dehydration) under drought stress conditions enhances tolerance (Shin et al., 2011). Induction of protein phosphatase 2C gene was positively associated with yield maintenance under drought with drought tolerant cultivars expressing higher levels of DREB transcription factor (Schafleitner et al., 2007b,c).

**Table 2:** **List of validated (RT-PCR and microarray) up-regulated (+) and down-regulated (−) genes implicated in drought stress response in potato**.

**Stress condition**	**Source**	**Gene family (gene)**	**Regulation**	**References**
PEG	Cell	AP-1 complex (γ-adaptin), catalase isozyme 1 (AW906659), sucrose synthase 2 (BE471969), LRR- receptor kinase (BF188424), hydroxyproline-rich extension (BF188513), protein heparanase (BF188529), 14-3-3 protein family (BE471953)	+	Ambrosone et al., 2011
Water	Leaf	Sucrose synthase genes	+	Evers et al., 2010
PEG	Leaf	GTP-binding proteins (BE341142)	+	Ambrosone et al., 2011
PEG	Cell	Sphingolipid protein membrane (Serinc)	−	Ambrosone et al., 2013
PEG	Cell	Phe ammonia-lyase (*PAL1*), Peroxidase (*Peroxidase 17*), sphingolipid protein membrane (Sphingolipid Desaturase), ubiquitin-conjugating Enzyme (ubiquitin-conjugating enzyme *E2*), ribosomal RNA components (*ribomosal L41,18s rRNA*), heat shock proteins (*HSP 20.2, HSP 83*),	+	Ambrosone et al., 2013
Water	Leaf	Elongation factor (*EF1*α)	+	Kondrak et al., 2011
Water	Leaf	Ubiquitin-proteasome protein (*UBC2*)	+	Kondrak et al., 2011
Water	Leaf	Thaumatin protein (*PGSC0003DMG400003569*), low-temperature-induced 65 kDa protein (*PGSC0003DMG400014293*), jasmonate ZIM-domain protein 1(*PGSC0003DMG400002930*), ethylene-responsive late embryogenesis (*PGSC0003DMG400000066*), ascorbate peroxidase (*PGSC0003DMG401001731*), WRKY transcription factor 4 (*PGSC0003DMG400009051*), non-specific lipid-transfer protein (*PGSC0003DMG400032250*), auxin repressed/dormancy associated protein (*PGSC0003DMG400012826*), early-responsive to dehydration 7 (*PGSC0003DMG402006194*), gibberellin 2-oxidase (*PGSC0003DMG400009021*), ethylene-responsive transcription factor (*PGSC0003DMG400014240*), ERF114 MYB transcription factor (*PGSC0003DMG400022399*), hydroxyproline-rich glycoprotein GAS31(*PGSC0003DMG400031105*),	+	Zhang et al., 2014
Water	Leaf	Germin (*PGSC0003DMG400014027*), abscisic acid receptor PYL4(*PGSC0003DMG400011033*), polygalacturonase-1 non-catalytic subunit beta (*PGSC0003DMG400027019*), transcription factor bHLH49 (*PGSC0003DMG400019659*)	−	Zhang et al., 2014
Water	Unspecified	MYB-like transcription factor (*StMYB1R-1*), protein 2 (*RD28*), aldehyde dehydrogenase (*ALDH22A1*), early responsive to dehydration (*ERD15-like*), homeodomain-leucine zipper (HD-Zip) proteins (*AtHB-7*)	+	Shin et al., 2011
Water	Leaf	delta 1-pyrroline-5-carboxylate synthase (AtP5CS),	+	Schafleitner et al., 2007a; Evers et al., 2010
Water	Leaf	Proline dehydrogenase (*PDH*)	+∕−	Schafleitner et al., 2007a
Water	Leaf	Galactinol synthase, arginine decarboxylase, spermidine synthase, proton gradient regulation 5	+	Evers et al., 2010
Water	Leaf	Spermine synthase, raffinose synthase	+∕−	Evers et al., 2010
Water	Leaf	Chaperone Dna K, thioredoxin	+∕−	Vasquez-Robinet et al., 2008
Water	Leaf	Ppiase Chl, Heat shock protein (*ER HSP 110 and ER HSP 90*)	+	Vasquez-Robinet et al., 2008
Water	Leaf	Glutathione-S-transferase, glutathione synthetase, Chaperone DnaJ	−	Vasquez-Robinet et al., 2008
Water	Leaf	chlorophyll a-b binding proteins, fructose-1,6-bisphosphatase, trehalose-6-phosphate synthase	−	Kondrak et al., 2012
Water	Leaf	MADS-box proteins (*AGL8 and AGL24*), GATA factor, Alfin 1 TF	−	Kondrak et al., 2011
Water	Leaf	*SU11B*, Rubisco small subunit (*RBCS-3B*)	+	Kondrak et al., 2011

*PEG, Polyethylene glycol; +, up regulation;−, down regulation; +∕−, up and down regulation*.

Constitutive overexpression of the *ScCBFI* gene from *Solanum commersonii* in transgenic *Solanum tuberosum* and *S. commersonii* plantlets grown *in vitro* showed better overall plant growth and root development under drought stress, providing evidence for a drought adaptation response (Teresa Pino et al., 2013). Transgenic potato plants that overexpress the *Arabidopsis thaliana* DHAR gene (*AtDHAR1*) in the cytosol show greater shoot extension than the WT plants due to elevated AsA (Ascorbate) leading to enhanced tolerance to drought and salt stress (Eltayeb et al., 2011). TPS1-expressing (Trehalose 6 phosphate synthase gene) transgenic potato lines effectively retain water under drought treatment and maintained an acceptable level of photosynthetic processes for a longer time than wild type as the transgenic lines had lower CO_2_ fixation rate and stomatal densities under optimal growth conditions than the non-transformed control plants (Stiller et al., 2008). The studies discussed above show that gene expression response under stressful conditions strongly depends upon the genotypic variation.

### Stress combinations

Plants interact with both abiotic (temperature, drought, and salinity) and biotic (herbivores and pathogens) factors either individually or in combination. Combination of stresses alter metabolism in ways that may differ from responses to different stresses applied individually (also dependent on species), as molecular signaling pathways controlling abiotic and biotic stresses may interact or counteract one another (Rizhsky et al., 2004; Mittler, 2006). As for abiotic stresses, changes in temperature, nutrient and the presence of heavy metals, toxins, and oxidants have the potential to contribute to plant stress and some of the aforementioned stress combinations can cause cell injury and produce secondary stresses such as osmotic and oxidative ones (Wang et al., 2003) with negative effects on yield, tuber quality, and market value (Wang-Pruski and Schofield, 2012; Rykaczewska, 2013). In *Arabidopsis*, transcriptome and metabolic profiling of plants subjected to a combination of drought and heat stress showed a partial combination of two multi-gene defense pathways and an accumulation of sucrose and other sugars such as maltose and glucose (Rizhsky et al., 2004). Evidence of evolutionary conservation of stress responses to salt, osmotic, heat, and ABA stimuli has been highlighted by Massa et al. (2013). Net photosynthesis and respiratory potential were lower in drought exposed plants 4 weeks after treatments [well watered and drought and with and without selenium (Se) foliar spraying] with Se inducing high respiratory potential in the leaves (Germ et al., 2007). Higher efficiency of energy conversion in PSII, expressed by higher quantum yield was observed in Se treated plants 2 weeks after treatment (Germ et al., 2007). Water deficit coupled with silicon (Si) application decreased total sugars and soluble protein concentration in potato leaves, with Si concentrations increasing in potato leaves under deficit conditions (Crusciol et al., 2009). Exposure of SO_2_ under well watered conditions produced defoliation and dry weight reduction of leaf, stem, and tubers while there was usually no dry weight reduction induced by SO_2_ under water stressed condition (Qifu and Murray, 1991). Well watered plants accumulated significantly higher leaf sulfur than did water-stressed plants at the same concentration of SO_2_ (Qifu and Murray, 1991). The combined effect of nematode infection and water stress resulted in decreased potato tuber production with reduced uptake of total P, K, and Mg (Fatemy and Evans, 1986; Fasan and Haverkort, 1991; Haverkort et al., 1991). As the interaction between stresses sometimes are regulated by the same signaling and regulatory networks, therefore, understanding these regulators connecting different response pathways provide better opportunities to breed for stress tolerant crop/genotypes covering broad spectrum.

## Potential traits for drought tolerance breeding in potato

An ability to maintain economic yield under water deficits is a valuable trait whenever water availability is a problem. This can be achieved by improvements in dehydration avoidance, dehydration tolerance and in other traits linked to optimum growth and metabolism under stress (Okogbenin et al., 2013). Desirable drought phenotypic traits must be genetically associated with yield under stress, highly heritable, genetically variable, easy to measure, stable within the measurement period, and must not be associated with a yield penalty under unstressed conditions (Okogbenin et al., 2013). Potato yields depend on the timing of water stress within the growing period (Spitters and Schapendonk, 1990) and upon climatic and soil conditions (Tourneux et al., 2003a) and thus it is necessary to consider these factors before making recommendations of optimal phenotypes for any specific environments. One might expect some characters such as enhanced WUE or improved tolerance of given water deficits to be generally adaptive. This is not generally the case as there will usually be some corresponding cost (e.g., higher WUE usually comes at the expense of lower photosynthetic rates while tolerance of water deficits often has a metabolic cost). Indeed the important character is not WUE but the “effective use of available water” (i.e., tailored to the specific growing environment) (Blum, 2009). For this reason results from artificial (e.g., hydroponic or controlled environment) studies may not often have direct relevance to drought tolerance in the field, though they may help to identify genotypes with characters predicted to be of value in specific environments (e.g., low stomatal conductance for consistently water-limited environments). We discuss below a number of traits which have been suggested as being crucial contributors to the genetic improvement of drought tolerance. While highlighting these traits, we also want to highlight that success rate of recovery after re-watering also depends upon the pre-drought intensity, duration, and species (Xu et al., 2010). In addition the species specific variation in hydraulic potentials is critical in steering the dynamic response of plants during recovery (Blackman et al., 2009).

### Stomatal characters

Plants adapt to drought conditions either by decreasing water loss or by maintaining water uptake. OA results in an increase in solutes (organic solutes and inorganic ions) in plant cells leading to a lowered osmotic potential, which in turn can improve cell hydration, help maintain cell turgor in leaf tissue, maintain metabolic processes and thus enhance plant growth and yield under drought stress (Morgan, 1984; Ludlow and Muchow, 1990; Sanders and Arndt, 2012). Studies on the diploid mutant “droopy” in potato whose leaves wilt during day leading to premature leaf fall indicates the advantage of stomatal hydrostat in normal plants as wilting and tall and slim structure of “droopy” was not attributed to root or vascular system but stomatal opening. Interspecific differences occur in species in their response and relationship of stomatal conductance to leaf water potential as stomatal conductance is controlled by complex interaction of intrinsic and extrinsic factors and not soil water availability alone. Nevertheless, studies mainly show that stomata close with increasing drought. Therefore, measuring stomatal characters (size and frequency) and control of water loss can aid in identification of desirable genotypes. Stomatal size and frequency are factors which influence stomatal resistance since most of the water escape through the stomata (Wang and Clarke, 1993). Remote sensing techniques using the thermal range of the spectrum can be used as an indication for plant water status and early studies showed the relationship between canopy temperature measured (indirectly influenced by stomatal closure) and plant water status in potato (Dallacosta et al., 1997; Leinonen et al., 2006). Thus differences in canopy temperature can be used as a selection method for screening drought tolerance among potato genotypes (Prashar et al., 2013; Prashar and Jones, 2014). These screening methods can be used to phenotype large populations to identify chromosomal regions controlling stomatal opening and closing and toward breeding crops with optimal stomatal response with some plasticity in behavior, so that stomata remain open under ample water conditions but close as water deficits increase.

### Photosynthesis

One of the primary physiological impacts of crop water scarcity is reduction of photosynthetic rate per unit leaf area (Moorby et al., 1975; Vos and Oyarzun, 1987; Schapendonk et al., 1989; Dallacosta et al., 1997; Deblonde and Ledent, 2001; Germ et al., 2007). The decreased net photosynthesis as a consequence of stomatal closure (Vos and Oyarzun, 1987; Trebejo and Midmore, 1990; Haverkort et al., 1991; Jefferies, 1994; Liu et al., 2005) and leaf area reduction under water stress are key contributors to yield loss under drought (Legay et al., 2011). Photosynthetically active radiation in plants is absorbed by chlorophyll and accessory pigments of chlorophyll-protein complexes which migrate to the reactive centers of PS I and II, where conversion of the quantum photosynthetic process takes place (Horton et al., 1996). Chlorophyll fluorescence helps in unraveling the different functional levels of photosynthesis including processes at the pigment level, primary light reactions, thylakoid electron transport reactions, dark enzymatic stroma reactions, and slow regulatory processes (Smillie and Nott, 1982; Maxwell and Johnson, 2000; O'Neill et al., 2006). The analysis of chlorophyll content and chlorophyll fluorescence parameters including initial fluorescence (*F*_0_), maximal fluorescence (*F*_m_), variable fluorescence (*F*_v_), and maximal quantum efficiency of PSII (*F*_v_/*F*_m_) is considered an important approach for the evaluation of health or integrity of the internal apparatus during photosynthetic processes within a leaf while providing a platform for rapid and precise detection and quantification of plants tolerance to drought stress (Vertucci et al., 1985; Chaerle and van der Straeten, 2000; Clavel et al., 2006; Buerling et al., 2013). These parameters might estimate influence of stress on growth and yield, since these traits are closely correlated with the rate of carbon exchange (Fracheboud et al., 2004; Czyczylo-Mysza et al., 2011). They can serve as reliable indicators to evaluate the energetic and metabolic imbalance of photosynthesis and yield performance across genotypes under limited water/stress scenarios (Araus et al., 1998). Components of the photosynthetic apparatus could be damaged in drought sensitive genotypes while drought tolerant genotypes can decrease or evade impairment resulting from drought stress (Smillie and Nott, 1982; O'Neill et al., 2006). Genetic differences in photosynthetic capacity exist at intraspecific and interspecific levels, so the use of the photosynthetic capacity as a physiological marker could be realistic where a positive relationship between the photosynthetic performance and the growth under stress conditions has been confirmed (Ashraf and Harris, 2013).

Chlorophyll fluorescence provides rapid indicators and a method for the study of changes in photosynthetic capacity of potato under water stress (Anithakumari et al., 2012) while the ratio between *F*_v_ and *F*_m_ and the differences in the canopy temperature can be used as a selection method for screening drought tolerance among potato genotypes (Ranalli et al., 1997; Prashar et al., 2013; Prashar and Jones, 2014). Chlorophyll fluorescence parameters *F*_0_, *F*_m_, *F*_v_, and *F*_v_/*F*_m_ show genotypic variation under drought stress in potato with drought stress having a reducing affect (Anithakumari et al., 2012). This is as a result of photoinhibition (Baker and Horton, 1987), reversible photoprotective down regulation or irreversible inactivation of PSII (Baker and Bowyer, 1994; Long et al., 1994). Water stress severity increase leads to a decrease in the heritability of *F*_v_/*F*_m_ (Jefferies, 1992; Zrùst et al., 1994; Anithakumari et al., 2012). There is evidence of a highly significant correlation between fluorescence emission and tuber yield with genotypes varying in response (Ranalli et al., 1997). A small decrease in quantum yield as a result of drought is associated with drought tolerance and this has been observed in early maturing varieties (Van Der Mescht et al., 1999; Anithakumari et al., 2012). Chlorophyll fluorescence parameters has the potential of separating genotypes according to their tolerance to water deficit (Newell et al., 1994).

Differences in leaf angle distribution can in principle affect light interception and photosynthesis (Jones, 2014), for example a theoretical analysis has shown that an increase in canopy leaf angle from 30 to 60° should result in a potential increase in rate of dry matter accumulation of between 15 and 30% after complete leaf-area expansion in maize (Tollenaar and Bruulsema, 1988). A similar morphological shift could be considered for potato by breeding for more erect leaved canopies. It could be hypothesized that genotypes which maintain canopy expansion and maximum light interception will yield higher dry matter content and significant yield under limiting water (Frusciante et al., 1999). Although higher photosynthesis rates, and associated characters such as stay green, are associated with higher yields (Cattivelli et al., 2008), it does not necessarily mean that selection for high assimilation will improve drought tolerance, as such lines may use water faster and suffer more from drought.

### Water use efficiency (WUE)

The growth and yield model of Passioura (1996) describes crop yield as the product of water uptake (WU), water-use efficiency (WUE), and HI. Improvement in any one of these components, while maintaining the others constant, can increase yield, but interactions can occur with increases in WU, for example, tending to be associated with lowered WUE. This means that selection for any one of these components must take account of effects on others. At the physiological level, WUE may be defined as the ratio of photosynthesis to transpiration as well as simultaneous ratio of net carbon assimilation to water transpiration of the stomata from the leaf (Xu and Hsiao, 2004; Guo et al., 2006). The leaf transpiration efficiency is determined by cellular solutes, specific ions, pH, and ABA produced in the leaf or imported from the root (Blum, 2009). WUE at whole plant and crop level is relatively lower when compared to the leaf level because of water loss associated with non-photosynthetic process and respiratory carbon loss during conversion of initial photosynthate to biomass as well as fraction of total water inputs that is never taken up by the plant (Guo et al., 2006). The factors influencing plant transpiration and water use at the whole plant level under drought include growth duration (Mitchell et al., 1996), leaf permeability (Kerstiens, 2006), nocturnal transpiration (Caird et al., 2007), leaf desiccation (Blum and Arkin, 1984), leaf area and orientation (Xu et al., 2009), leaf growth (Weisz et al., 1994), and soil evaporation (Rebetzke and Richards, 1999). There is a tendency for WUE to increase as stomata close (Vos and Groenwold, 1989; Jones, 2014). The advantage of constitutive stomatal closure is expected to be only valid in consistently dry environments, where it can lead to enhanced WUE, but where drought occurrence is uncertain, constitutive closure is likely to be disadvantageous for yield unless genotypes show responsive flexibility in opening and closing dependent upon stress. In consistently dry environments, genotypes that can deplete soil moisture only slowly and may optimize crop WUE rather than maximizing short-term growth. Ideally they will continue until the soil water is depleted, at which point growth is halted (Elsharkawy et al., 1992). One might expect plants with higher stomata number per unit area, or with greater length and width, to lose more water, but there may be natural compensations between size and frequency (Jones, 1977), while stomatal movements can override such morphological differences (Jones, 1987). A low rate of cuticle transpiration may reduce leaf dehydration and promote leaf survival (Wang and Clarke, 1993). Early vigor has the potential of improving both WU and WUE, while deep roots and/or osmoregulation under appropriate conditions increase water extraction from the soil (Richards, 2006; Blum, 2011; Sadok and Sinclair, 2011).

Significant progress in assessing genetic variability in WUE was achieved after the establishment of the physiological links between δ^13^C and WUE (Farquhar and Richards, 1984; Farquhar et al., 1989). δ^13^C has been used as a surrogate for WUE and has been successfully used for tomato (Martin and Thorstenson, 1988), wheat (Rebetzke et al., 2002), and rice (Impa et al., 2005). Nevertheless, although understanding the inheritance of δ^13^C could be useful for the development of potato cultivars with high WUE (Anithakumari et al., 2012), an understanding of the relationship between TE, WUE and yield under different levels of water stress is important. Improvement of the WUE of a crop plant results in higher yield performance if high HI can be maintained as total biomass yield in drought environment is positively associated with WUE (Blum, 2009; Araus et al., 2012). TE is under genetic control (Masle et al., 2005) and excludes amount of water lost by soil evaporation, and hence should be considered as a potential trait for drought stress (Manavalan et al., 2009). It should be that WUE can primarily be the result of limited water use rather than a net improvement in plant production or assimilation and therefore for selection trait evaluation both for resource efficiency and final product is crucial. An effective breeding strategy will consider plant adaptive characteristics which drive effective use of water, resultant dehydration avoidance and yield potential (Blum, 2009).

### Maintenance of water status: canopy temperature and development

Plant water status maintenance is possible if the plant is equipped with the appropriate “hydraulic machinery” (Sperry et al., 2002) as well as additional traits to relieve the energy load on the plant as well as manage an effective use of water (Chaves et al., 2002; Blum, 2011). The state of water flux, transpiration, and the associated leaf water potentials can be used to comparatively phenotype toward identification of drought tolerant genotypes. The obvious limitation is the dynamic nature of the inherent traits modulating water status during the day. With a robust and automated phenotyping platform this problem can be circumvented by “dawn to dusk” measurements. Plants that can maintain adequate relative water content (RWC) for a longer period of time under drought exposure will have the greatest likelihood of continued metabolic functioning and survival and studies have been able to demonstrate that cultivars with greater drought resistance were able to maintain higher cellular hydration under drought conditions (McCann and Huang, 2008). The RWC has successfully been used in differentiating drought resistance and drought susceptible potato cultivars (Coleman, 1986). A relatively lower canopy temperature in drought stressed crop plants indicates maintenance of high stomatal conductance; this can indicate effective maintenance of tissue water status particularly by effective uptake of soil moisture or by other adaptive traits (Blum, 2009). Studies using various crops including wheat (Amani et al., 1996; Rebetzke et al., 2013), rice (Horie et al., 2006), sorghum (Mutava et al., 2011), and more recently potato (Prashar et al., 2013) have all reported that canopy temperatures can be associated with yield and could therefore be used as a selection technique (Jackson et al., 1981; Leinonen and Jones, 2004; Grant et al., 2007; Jones, 2007; Zia et al., 2013). Therefore, a better understanding of the mechanisms that regulate growth under water deficit, such as those involved in shutting down meristem activity will be vital in the development of new technologies to increase growth under stress (Tisne et al., 2010).

### Root characters

The morphology and architecture of the root system at any stage of development may influence the hydrostatic gradient. The resistance in the root system to water flow both radially (into the root) and axially (within the xylem) may be great enough to increase significantly the gradient, reduce the hydraulic conductivity and increase the canopy temperature (Mahan et al., 1995). Potato is very sensitive to water-stress when compared to other species (Porter et al., 1999) because of its shallow (Iwama and Yamaguchi, 2006) and sparse (Jefferies, 1993a) root system. The tendency for an increased root—shoot ratio under drought and for root growth to be maintained more than shoot growth (Jefferies, 1993a) are both likely to contribute to drought tolerance.

Breeding of new cultivars with excellent root quality that ensures absorption of water from deeper soil layers and under low soil moisture will help in more efficient utilization of water for potato production (Iwama, 2008). Differences in gross morphology, including the degree of branching and rooting depth may decrease the availability of water due to the inability of the root system to explore a larger soil volume and thus increase the gradient (Mahan et al., 1995). Genotypic differences have been reported for both rooting depth (Steckel and Gray, 1979; Tourneux et al., 2003a,b) and root growth volume (Mackerron and Peng, 1989; Jefferies, 1993a), while the positive correlation between root mass, shoot mass and final tuber yield have led to the suggestion of using root mass in the plow layer as a selection criterion (Iwama, 2008). One approach to the selection of deep rooting genotypes is to measure the pulling resistance (PR) of roots (Ekanayake and Midmore, 1992; Stalham and Allen, 2004). Differences in the ability of roots to continue to elongate under low water potential could also be an adaptation to water deficit (Westgate and Boyer, 1985; Spollen et al., 1993) as would more efficient rooting architectures (Porter and Semenov, 2005; Tardieu, 2012; Wishart et al., 2013). Early vigorous root proliferation may be a useful selection trait for maintaining yield of potato under restricted water level (Puértolas et al., 2014). It is noteworthy to mention that the concept of root ideotype can only be exploited in practical field breeding with a thorough knowledge of the plants stress environment as well as the metabolic cost sustained by the plant to develop and maintain a more vigorous root system (Tuberosa, 2012). In addition to well recognized factors of root density and depth, the hydraulic characteristics of the plant, and its interaction with the soil environment is highly significant in drought adaptation (Vadez, 2014).

### Metabolites and biochemical response

OA is a key drought adaptive mechanism that enables plants to maintain water absorption and cell turgor pressure and thus potentially contribute to sustained higher photosynthetic rate and expansion of growth (Cattivelli et al., 2008). The accumulation of a metabolite during drought does not functionally link with an increase in the tolerance level or with tolerance differences between genotypes. Metabolite levels can increase due to increasing degradation or a reduced biosynthesis of another metabolite without any protective effect (Degenkolbe et al., 2013). The response at metabolic level varies between genotypes and studies have shown differential accumulation of osmotically active solutes. This response to stress has also been used as an indicator of drought tolerance in various model plants and crop species (Schafleitner et al., 2007c; Vasquez-Robinet et al., 2008; Evers et al., 2010). Studies in potato have demonstrated that drought stress leads to accumulation of osmotically active solutes including proline, inositol, raffinose, galactinol, and trehalose (Vasquez-Robinet et al., 2008; Evers et al., 2010; Legay et al., 2011; Kondrak et al., 2012) indicating an osmoprotector or osmoregulatory function (Schafleitner et al., 2007a; Teixeira and Pereira, 2007). Proline serves as an ROS scavenger and is used as a non-enzymatic antioxidant to counteract the damaging effect of different ROS members helps in water stress survival through osmoprotection (Vanková et al., 2012). Proline synthesis and catabolism are required for optimal growth at low water potential while its metabolism and function in maintaining a favorable NADP/NADPH ratio are relevant to understanding metabolic adaptations to drought and efforts to enhance drought resistance (Sharma et al., 2011). Proline can act as a signaling molecule to modulate mitochondrial functions, influence cell proliferation or cell death and trigger specific gene expression, which can be essential for plant recovery from stress (Szabados and Savouré, 2010). Similar to metabolic, biochemical response also changes as a function of stress. Win et al. (1991) reported that application of antitranspirants that raised leaf water potential and altering leaf: tuber potential gradients in potato plants subjected to water stress led to greater Ca^2+^ accumulation in tubers and reversed Ca^2+^ deficiency-related tuber necrosis. Studies by Lefevre and colleagues showed an increase in the concentration of the majority of analyzed cations in a large number of cultivars in response to water deficits while identifying two cultivars that were able to maintain good yield stability in association with high mineral content under water deficit (Lefevre et al., 2012). Biochemical responses of potato to drought are complex with levels of antioxidants showing increases, decreases, or no effect, depending on the genotype and kind of antioxidant (Andre et al., 2009; Wegener and Jansen, 2013). An increased activity of peroxidase, superoxide dismutase and catalase in response to oxidative stress has been highlighted in potato (Boguszewska et al., 2010) but antioxidant contents of yellow tuber bearing cultivars are weakly affected by the drought treatment as compared to non-yellow tuber bearing types which show high cultivar-dependent variations (Andre et al., 2009).

### Harvest index

HI is a key partitioning index that shows the extent of remobilization of photosynthates to tubers. Studies recommend that maintaining a high HI is the best strategy for improving crop yields under drought stress conditions (Ludlow and Muchow, 1990). Different adaptive mechanisms may associate with growth, biomass partitioning and yield under variable drought stress conditions (McClean et al., 2011). Although, the contrasting responses of the genotypes in response to drought may reflect very distinct evolutionary strategies, change in partitioning to plant parts during early stage of growth is considered as an adaptive response to drought stress of resistant genotypes (Specht et al., 2001; McClean et al., 2011). High HI coupled with high leaf/stem ratio with low number of branches may contribute to achieve high and stable potato yields in drought prone environments (Iwama, 2008; Deguchi et al., 2010) and similarly, identifying genotypes that may use photosynthates for greater tuber expansion at the expense of shoot biomass will help in drought tolerance selection.

## Future research perspective

Breeding for drought tolerance presents a challenge as it is a genetically complex polygenic trait with multiple pathways implicated. Success in this objective not only helps in extending the cultivation of crops into drought prone areas but in addition may allow more stable yield under environmental fluctuations. Identifying genetic variation for drought tolerance is the first basic requirement for breeding advancement under drought. An understanding of the genetic architecture of drought resistance components is an important milestone. Effective crop improvement for drought tolerance will require the pyramiding of many disparate characters, with different combinations being appropriate for different growing environments. The difficulty of pyramiding drought tolerance related genes in highly heterozygous tetraploid potato cultivars while considering other important economic traits presents a major challenge with linkage drag and distortion in segregation between inter-specific hybrids presenting further challenges. Hopefully these issues can be circumvented by using biotechnological approaches in future years.

**Figure 3:**
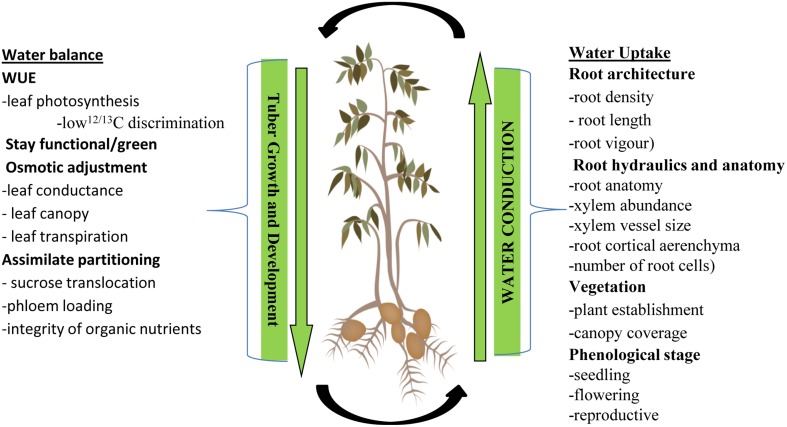
**A hypothetical model of morphological and physiological traits implicated during water uptake and balance in drought stressed potato**.

Wild species and adapted germplasm are the reservoir of many useful genes/alleles as they have evolved under natural selection to survive climate extremes (Sharma et al., 2013) and thus evaluation of this material is essential for progress. Considerations should be made in respect to timing and intensity of drought (Chaves et al., 2003; Cattivelli et al., 2008; Coleman, 2008). An understanding of the interaction between below ground water uptake by the roots and above ground water loss from the shoot system is essential and helpful for breeding under different environment scenarios. Figure 3 presents the summarized information on potential adaptive traits under water stress. These traits are principally involved in water uptake and energy translocation and we hold the view that for sustainable management of water stress effect combination of these traits would help in developing stress tolerant varieties in potato. It is crucial to understand the interaction between these agronomical, physiological and morphological traits in order to understand drought tolerance and breed toward sustainability.

An integrated approach is needed where high throughput genotyping can be precisely linked with phenotyping under different field environmental conditions. A lack of precision phenotyping is creating a significant bottleneck to progress. Recent advancement in imaging sensor technology has made it feasible to evaluate stress related traits under field conditions remotely. Infra-red thermography (IRT) has been used as a phenotypic resource for evaluating plant stress (Jones et al., 2009; Prashar et al., 2013) and this needs to be explored using multi-sensor approaches (Furbank and Tester, 2011; Araus and Cairns, 2014). Efficient use of thermography for high-throughput phenotyping (HTP) demands adequate control of variation resulting from the environment and the use of appropriate normalization techniques (Prashar et al., 2013; Prashar and Jones, 2014). Although IRT combined with effective image analysis provides a powerful approach for comparing large number of genotypes for field scale phenotyping of characters related to plant water relations, a single sensor approach has limitations as complex stress traits are not just influenced by one physiological or morphological component. With IRT providing information on stomatal changes, reflecting WUE, and crop productivity, when used in combination with other available technologies such as fluorescence and hyperspectral imaging it can be more powerful (Chaerle et al., 2009; Furbank and Tester, 2011; Mahlein et al., 2012; Busemeyer et al., 2013; Andrade-Sanchez et al., 2014; Luis Araus and Cairns, 2014) in understanding complex traits such as drought (Topp et al., 2013; Honsdorf et al., 2014). High throughput phenotyping also allows the comprehensive assessment of complex traits by allowing quantitative measurements of the contribution individual parameters. With the new technological advances in HTP methods assessment of three-dimensional characters like plant architecture or plant shape is becoming feasible. As discussed previously, plant architecture and its development provides useful information on plant stress responses and adaptation and their association with yield, thus enabling the linking of high throughput genotyping with high throughput phenotyping both for above and below ground characteristics (Dhondt et al., 2013; Chen et al., 2014; Deery et al., 2014; Paulus et al., 2014).

Along with effective analysis and reproducibility for high throughput phenotyping, the other major requirement for drought field phenotyping is to have adequate water stress conditions and achieving proper control over the field stress environment in order to assure relevant drought test profile (Tuberosa, 2012). It is therefore imperative that researchers should select ideal drought prone regions with limiting rainfall or irrigation distributed over a reasonable time frame. Artificial drought control with rain-out shelters can also be helpful. Experimental protocols involving field and control environment should complement each other as some physiological traits are better exploited in either situation. The importance of understanding the replication and repeatability of the results under field conditions is essential to characterize the effects of QTLs and to evaluate stability in diverse environment and breed for different conditions and different scenarios of stress interaction (Cattivelli et al., 2008). Analysis of G × E (genotype × environment), which is becoming very common, should be incorporated with management (M) in any controlled and field experiment for identifying genotypes for specific environment and under specific management practices system (G × E × M) to overcome issues relating to stress traits.

The candidate gene strategy bridges the division between quantitative and molecular genetics in studying complex drought stress responses and has been gainfully applied to rice and barley drought tolerance (Nguyen et al., 2004; Tondelli et al., 2006). Comparative *omics* analysis on stress-responsive epigenomes could aid in understanding of drought adaptations. Transgenic research effort should focus on conferring drought tolerance while increasing or stabilizing tuber yield. This implies that drought transgenics should have extensive root system, reduced stomata density and high WUE while possessing higher levels of ABA, proline, soluble sugar, reacting oxygen species-scavenging enzyme activities during drought stress (Werner et al., 2010; Yu et al., 2013). Transgenic approaches should play a role in the future in developing drought tolerant potato cultivars with incorporation of specific cloned genes and restricting the transfer of undesirable genes from donor organism (Ashraf, 2010). Similarly, use of emerging genome engineering techniques like a RNA-guided endonuclease technology for sequence-specific gene expression known as clustered regularly interspaced short palindromic repeats (CRISPR) provides a simple approach for selectively perturbing gene expression on a genome-wide scale (Sander and Joung, 2014). As stress traits are complex traits, approaches like gene pyramiding not only for stress combinations but also specific stress can be targeted with cautious (Ashraf, 2010). This approach has led to engineering genes that encode compatible organic osmolytes, plant growth regulators, antioxidants, heat shock proteins, and TFs involved in gene expression in potato (Stiller et al., 2008; Shin et al., 2011; Zhang et al., 2011; Kondrak et al., 2012; Cheng et al., 2013; Pal et al., 2013; Pieczynski et al., 2013). Transgenics can be used to their full potential with the lifting of legislative barriers in many countries so as to provide opportunities for extensive field testing in different environments.

The plant root system can act as “resource island” which attracts and selects specific microbial communities that can promote plant growth through enhancement of plant photosynthetic activity and biomass synthesis under water deficit (Ruiz-Sanchez et al., 2011; Marasco et al., 2012; Naveed et al., 2014). Studies have demonstrated the potential of *arbuscular mycorrhizal* (AM) symbiosis in developing drought tolerant maize and sweet potato cultivars respectively (Boomsma and Vyn, 2008; Naher et al., 2013). AM enhanced the adaptation of sweet potato plants to drought stress as indicated by higher values of leaf RWC, root dry weight, root length, transpiration, and WUE as compared to non-AM plants (Naher et al., 2013). These microorganisms offer great potential because the resulting tolerance/adaptability could be ascribed to changes in soil microbial community rather than genetic changes in plants. For example, soil microbes can prime plants to increase drought resistance compounds or elicitors more rapidly and thus increase resistance to stress (Horn et al., 2013; Okamoto et al., 2013). However, the ability of crops to take advantage of soil microorganism induced drought tolerance may be hindered by modern agricultural practices that reduce crop-soil interactions (Bennett et al., 2013).

A holistic approach involving morphological, physiological, biochemical, phonological, and anatomical responses to water stress conditions should provide the best opportunities for enhancing drought tolerance in the potato crop. Concerted efforts are required by geneticists, physiologists, breeders, agronomists, and technologists toward precise and accurate phenotypic evaluations and the development of platforms for the automated analysis of the necessarily large mapping populations. This will ensure good monitoring and phenotyping of physiological, morphological, and growth parameters under water stress. Identification of more potato phenotypes that correlate positively with drought performance in the field is urgently needed. It is noteworthy to mention that different environmental conditions in different potato growing regions demand diverse options and there is a lot to learn and gain from their interactions with the complex nature of the trait.

## Conclusion

One of the great challenges for the next decade is to mitigate any effect of climate change on crop production with a main focus being to maintain crop production levels with reduced availability of water. A multi-pronged approach using combined expertise will be critical in sustaining potato production. Efforts need to be intensified to improve the database of potato drought-related genes and our understanding of their potential roles in drought responses. Natural variation in wild and cultivated potato germplasm provides an excellent platform for the discovery of diagnostic markers for marker-assisted selection (MAS) and for cloning and insertion of drought resistance genes applicable to diverse agrarian zones. Although genetic manipulation, using key genes identified from functional studies, offers significant opportunities for the development of drought tolerant varieties it will be necessary to ensure that any deleterious negative effects are avoided. The emergence of novel approaches involving the use of high throughput “omics” technologies (also known as “Phenomics”) including genetic, physiological, biochemical, molecular and biotechnological techniques offer hope for exciting innovations toward maintaining food and income security, mitigation of poverty, and reduction of farmers' risk in vulnerable agricultural environments.

## Conflict of interest statement

The authors declare that the research was conducted in the absence of any commercial or financial relationships that could be construed as a potential conflict of interest.
